# Discovering cancer genes by integrating network and functional properties

**DOI:** 10.1186/1755-8794-2-61

**Published:** 2009-09-19

**Authors:** Li Li, Kangyu Zhang, James Lee, Shaun Cordes, David P Davis, Zhijun Tang

**Affiliations:** 1 Department of Bioinformatics, Genentech Inc., 1 DNA Way, South San Francisco, CA 94080, USA; 2 Department of Molecular Biology, Genentech Inc., 1 DNA Way, South San Francisco, CA 94080, USA

## Abstract

**Background:**

Identification of novel cancer-causing genes is one of the main goals in cancer research. The rapid accumulation of genome-wide protein-protein interaction (PPI) data in humans has provided a new basis for studying the topological features of cancer genes in cellular networks. It is important to integrate multiple genomic data sources, including PPI networks, protein domains and Gene Ontology (GO) annotations, to facilitate the identification of cancer genes.

**Methods:**

Topological features of the PPI network, as well as protein domain compositions, enrichment of gene ontology categories, sequence and evolutionary conservation features were extracted and compared between cancer genes and other genes. The predictive power of various classifiers for identification of cancer genes was evaluated by cross validation. Experimental validation of a subset of the prediction results was conducted using siRNA knockdown and viability assays in human colon cancer cell line DLD-1.

**Results:**

Cross validation demonstrated advantageous performance of classifiers based on support vector machines (SVMs) with the inclusion of the topological features from the PPI network, protein domain compositions and GO annotations. We then applied the trained SVM classifier to human genes to prioritize putative cancer genes. siRNA knock-down of several SVM predicted cancer genes displayed greatly reduced cell viability in human colon cancer cell line DLD-1.

**Conclusion:**

Topological features of PPI networks, protein domain compositions and GO annotations are good predictors of cancer genes. The SVM classifier integrates multiple features and as such is useful for prioritizing candidate cancer genes for experimental validations.

## Background

Cancer is a complex disease whose multi-step progression involves alteration of many genes, including tumor suppressor genes and oncogenes. Although multiple targeted cancer therapeutic agents have been developed based on several known cancer genes, it is expected that many cancer genes remain to be identified [1]. Identification of novel genes likely to be involved in cancer is important for understanding the disease mechanism and development of cancer therapeutics. Recently, efforts in global genomic re-sequencing have been made to identify novel cancer genes by detecting somatic mutations in tumor tissues [2-4]. However, it is challenging to distinguish true cancer-associated mutations from a large amount of "passenger" variants detected in these studies that are likely to be irrelevant to cancer progression.

Most gene products interact in complex cellular networks. It was proposed that direct and indirect interactions often occur between protein pairs whose mutations are attributable to similar disease phenotypes. This concept was utilized to predict phenotypic effects of gene mutations using protein complexes [5] and identify previously unknown complexes likely to be associated with disease [6,7]. Similar notion may be applied to cancer where identifying protein interaction network of known cancer genes may provide an efficient way to discover novel cancer genes. The rapid accumulation of genome-wide human PPI data has provided a new basis for studying the topological features of cancer genes. It was shown that the network properties in human protein-protein interaction (PPI) data, such as network connectivity, differ between cancer causing genes [1] and other genes in the genome [8]. An interactome-transcriptome analysis also reported increased interaction connectivity of differentially expressed genes in lung squamous cancer tissues [9]. These studies indicated a central role of cancer proteins within the interactome. Recent studies also applied network approaches to studying cancer signaling [10] and identifying biomarkers of cancer progression in specific cancer types [11,12]. However, the utility of PPI network for identification of novel genes whose genetic alterations are likely to be causally implicated in oncogenesis remains to be demonstrated. In addition, efforts have been made to use functional and sequence characteristics, such as GO annotation and sequence conservation, to predict cancer genes and cancer mutations [13,14]. However, a systematic analysis of all these features side-by-side is needed to evaluate their merits, both individually and in combination, in cancer gene prediction.

In this study, we took a machine learning approach to investigate various network and functional properties of known cancer genes to predict the likelihood of a gene to be involved in cancer. Although Cancer Gene Census provides a catalogue of currently known cancer causing mutations, many other cancer genes may be yet to be discovered from the rest of the genome. To reduce the false positives in classifying genes not involved in cancer, we extended the comparison of various features in four non-overlapping gene groups, i.e. "cancer genes" from the Cancer Gene Census (*bona fide *cancer genes whose mutations are causally implicated in cancers) [1], "COSMIC genes" profiled for somatic mutations in cancer and deposited into the Catalogue Of Somatic Mutations In Cancer (COSMIC) database [15] (excluding those in the cancer gene set), "OMIM genes" from the Online Mendelian Inheritance in Man (OMIM) database [16] (excluding those in the cancer or COSMIC gene set), and other genes in the genome (noted as "non-cancer genes"). Somatic mutations were observed for a subset of "COSMIC genes" in cancers and they are potentially related to oncogenesis while "OMIM genes" contain known genes involved in diseases other than known cancer genes. We trained various classifiers using "cancer genes" and "non-cancer genes", and evaluated the contribution of various features and different classification methods using cross validation. We then applied the trained classifier with the best cross validation performance to human genes to prioritize human genes likely to be involved in cancer. To evaluate the roles of predicted cancer genes in cancer cell growth and proliferation, siRNA knock-down experiments and cell viability assays were conducted in human colorectal cancer cell line.

## Methods

### Datasets

PPI network was constructed as the union of all relationships obtained from representative published datasets [8,17,18]. Sequence features were obtained from NCBI Entrez database [19]. The number of alternative transcripts for each Entrez gene was obtained from the RefSeq database. Non-synonymous mutation rate Ka and synonymous mutation rate Ks of human-mouse and human-rat orthologs were retrieved from NCBI HomoloGene database .

We constructed four non-overlapping gene groups, i.e. "cancer genes" from the Cancer Gene Census [1], "COSMIC genes" from the Catalogue Of Somatic Mutations In Cancer (COSMIC) database [15] excluding genes in the cancer gene group, "OMIM genes" from the Online Mendelian Inheritance in Man (OMIM) database [16] excluding genes in the cancer gene group or COSMIC gene group, and rest of the genes (noted as "non-cancer genes").

From human genome, 9218 Entrez genes were mapped to all of the following datasets; PPI network, Refseq database, HomoloGene human-mouse and human-rat orthologs, GO annotations, and Pfam database. Among these, 278 belong to cancer, 2191 belong to COSMIC, 1088 belong to OMIM, and 5661 belong to non-cancer gene set, respectively.

### Enrichment of Pfam and GO

The Pfam or GO log-odds scores [14] were developed to represent the relative frequency of a Pfam protein domain [20] or a GO term annotated in cancer and non-cancer gene sets, respectively. For Pfam domain log-odds scores, boundary conditions were adopted to correct cases with no presence of a particular domain in the cancer or other gene groups (1.1). Only domains in the Pfam-A category were included to compute the Pfam log-odds score. The GO log-odds score were computed for each GO slim term. GO slim is simplified version of the GO ontology containing a subset of the terms in the whole GO. GO slim was used because it gives a broad overview of GO ontology content with reduced details. We used "Generic GO slim" annotations downloaded from the Gene Ontology website . For genes with multiple Pfam domains or GO terms, the log-odds scores were summed to obtain a log-odds score for each gene. For feature selection of GO terms and Pfam domains for the classifiers, chi-square tests were conducted using R  on the full set of GO terms and Pfam-A domains, which were then ranked by the p-values from chi-square tests. To remove redundancy in feature selection of GO terms, GO terms with less significant p-values than their parent terms were removed such that GO terms at higher hierarchy of the onotology and with more significant p-values were prioritized to be included as features.

(1.1)

### Training and evaluation of classifiers

SVM classifier was built using LIBSVM tools, a library for Support Vector Machines . SVMs were trained on cancer genes and non-cancer genes to estimate the probability of a gene to be involved in cancer. We chose radial basis function (RBF) as the kernel of SVM. We conducted cross-validation to select the parameter *gamma *for the radial basis function kernel and the parameter *c *for the cost of training error. Cost weights *wi *were set based on the ratio between the number of negative examples and the number of positive examples in the training data. Naïve Bayes and logistic regression classifiers were built using default parameters from Weka tools .

The features used to train the classifiers include degree, clustering coefficient and average length of shortest path to a cancer gene from the PPI network, gene and protein lengths from sequence features, Ka and Ka/Ks from evolutionary features, presence or absence of annotation of selected GO terms and Pfam domains (p < 0.01 from chi-square tests of over- or under-representation in cancer genes compared with non-cancer genes). Continuous features whose distribution deviates significantly from the normal distribution were log transformed, including PPI degree, protein length, gene length, Ka and Ka/Ks.

Classifiers were trained and evaluated using cancer genes as positive examples and non-cancer genes as negative examples. 10-fold cross validation experiments were conducted to evaluate the performance of the classifiers. The dataset was randomly divided into ten subsets, each of which has one tenth of the number of examples in the original set and preserves the relative proportion between positive and negative examples. A classifier was trained and tested ten times where each time a different subset was used for testing and the remaining nine subsets were used for training. For SVM classifiers, we conducted 5-fold cross-validation using the training data for each round to select parameter pair *c *and *gamma*, and then a classifier trained using the selected parameter pair was evaluated using the test data (parameter pair was selected from *c *= 1, 4, 16, 64 and *gamma *= 0.001, 0.01, 0.1, 1). The area under the ROC curve (AUC) was used to measure the performance of different classifiers. ROC curves and AUC values were obtained using the LIBSVM and Weka tools. A classifier with better performance than a random predictor has an AUC between 0.5 and 1.

### siRNA experiments

DLD-1 (ATCC CCL-221) cells were obtained from the American Type Culture Collection and maintained in High Glucose Dulbecco Modified Essential Media supplemented with 10% Fetal bovine sera and 2 mM L-Glutamine. Gene targeting siRNA duplexes (Dharmacon siGENOME), siGENONE Non-Targeting siRNA #2 (Dharmacon D-001210-02) and siGENOME Non-Targeting siRNA Pool #1 siRNAs were transfected into cells using Lipofectamine 2000 (InVitrogen #11668). siRNA duplexes were transfected at concentrations of 25-30 nM (duplexes) or 100-120 nM (pools), respectively. Lipid-siRNA complexes were formed in OptiMEM Media (Gibco #31985) to which cells were added in antibiotic-free media. Four days following transfection, cell viability was measured with the addition of CellTiter-Glo (Promega #G7570) and luminescence was measured according to manufacturer's instructions using a Perkin Elmer EnVision luminometer.

### Cell viability data analysis

A viability score is defined as the ratio of CellTiter-Glo readout between transfection of a testing siRNA and that of negative controls (non-targeting siRNAs). A viability score less than 1 indicates decreased cell viability with siRNA targeting a given gene. Viability scores for two replicated transfections using the same siRNA were averaged and statistical significance of reduced viability was evaluated by *t *tests for each siRNA oligo. As there are four siRNAs targeting a given gene, we require at least two of the siRNAs have p-value less than 0.05 and the decrease in cell viability is at least 15% to claim that the gene is essential for cell viability.

## Results

### Protein-protein interaction network and gene sets

A comprehensive human PPI network was built via integrating multiple publicly available data sources, including a collection of validated direct interactions [17], computationally predicted interactions based on homology mapping [8], and experimentally proposed interactions from large-scale human mass-spectrometry experiments [18]. Validated interactions were derived from the Biomolecular Interaction Network Database (BIND) [21], the Human Protein Reference Database (HPRD) [22], Reactome [23], and the Kyoto Encyclopedia of Genes and Genomes (KEGG) [24]. All proteins were mapped to Entrez [19] genes and the combined human PPI network consists of 13,802 genes (genes and their protein products are used interchangeably below) and 140,600 interactions. 331 out of 368 (89.9%) genes from Cancer Gene Census, 2769 out of 3,001 (92.3%) genes from COSMIC (excluding genes also in Cancer Gene Census), 1786 out of 1976 (90.4%) genes from OMIM (excluding genes also in Cancer Gene Census or COSMIC) and 8916 out of 18,744 (47.6%) remaining genes in the human genome were included in the PPI network.

To investigate various PPI network, functional and sequence features of cancer genes and other genes, we selected 9218 well-annotated genes that were included in the PPI network and were assigned with GO terms [25] and Pfam domains [20]. They also have mouse or rat orthologs defined by HomoloGene [26]. We classified these genes into four mutually exclusive sets, including 278 "cancer genes" from Cancer Gene Census, 2191 "COSMIC genes" from the COSMIC database (excluding those also in Cancer Gene Census), 1088 "OMIM genes" from the OMIM database (excluding those also in Cancer Gene Census or COSMIC), and the rest 5661 "non-cancer genes".

### Topological features in PPI network

**Figure 1:**
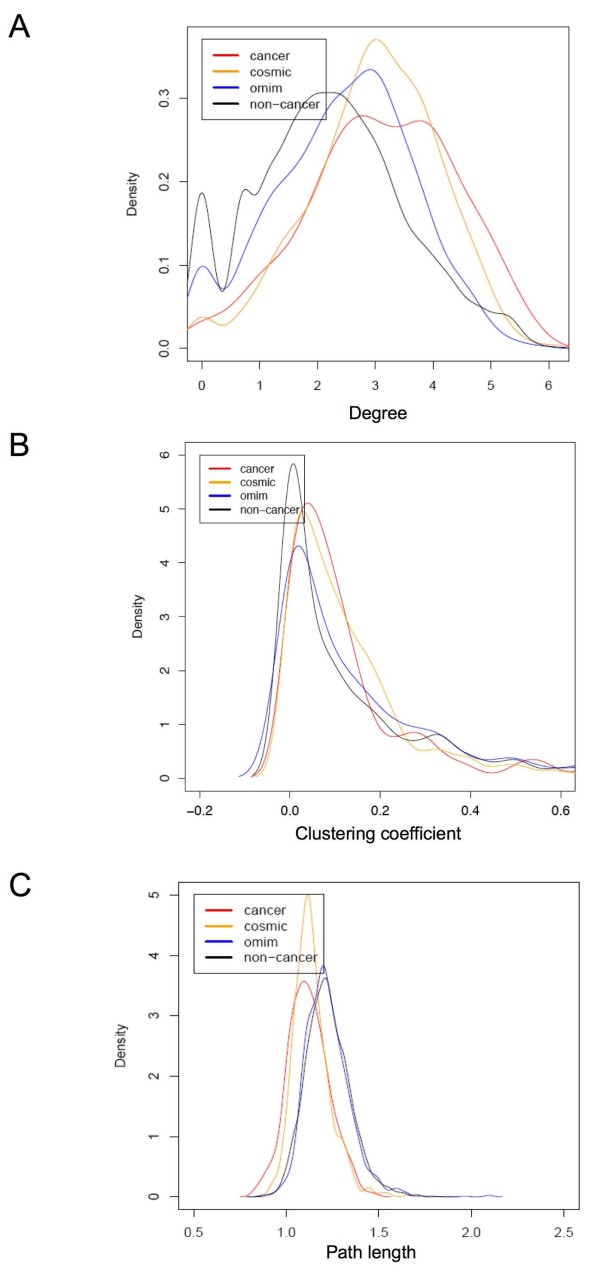
**Probability density distributions of PPI network topological features**. A) log(degree) B) clustering coefficient C) log(average path length towards cancer genes).

Three topological features of the PPI network were computed for each gene: the network connectivity, the clustering coefficient [27], and the average shortest path length towards a cancer gene. Comparisons of these network topological features among cancer, COSMIC, OMIM, and non-cancer genes show that COSMIC genes are similar to cancer genes. Pair-wise comparisons of cancer genes vs. non-cancer genes and cancer genes vs. OMIM genes show significant difference in all three topological features (Figure 1; see additional file 1: Supplementary Table S1). The network degree measures the number of interaction partners for a given gene. The network degree distributions are significantly different among the four gene groups (one-way ANOVA F-test p-value 1e-11, Kruskal-Wallis rank sum test p-value 0.0001). Cancer genes show the highest degree of interaction (median = 23), followed by COSMIC genes (median = 18), OMIM genes (median = 14) and non-cancer genes (median = 8) (Figure 1A). The clustering coefficient measures the network neighborhood interconnectivity for a given gene, and serves as an indicator of the network density in a gene's neighborhood community [27]. Cancer and COSMIC genes show higher clustering coefficient than OMIM and non-cancer genes (Figure 1B). This indicates that cancer proteins appear in a more densely connected network community than OMIM and non-cancer genes. The average shortest path length towards all cancer genes was selected to represent how close a gene is to cancer genes in the PPI network. As cancer progression is considered to be the result of deregulation of inter-related pathways, a gene will be more likely to be involved in cancer progression if it is close to known cancer genes in the PPI network. In fact, four gene groups exhibit distinctive distribution of the average shortest path length with cancer gene being the shortest, followed by COSMIC genes, OMIM genes and non-cancer genes (Figure 1C). The shorter average path length for COSMIC genes towards known cancer genes compared with OMIM and non-cancer genes may indicate that COSMIC genes are more likely to be involved in cancer related cellular pathways. The results demonstrated that all these network properties can contribute to identifying cancer genes and it is feasible to construct a classifier to combine these features to identify cancer genes. The similarity between COSMIC genes with cancer genes indicates that many COSMIC genes are likely to be putative cancer genes and should be distinguished from the non-cancer gene set. In addition, PPI network properties may be able to help distinguish these putative cancer genes from non-cancer genes. It is worth noting that although both "cancer genes" and "OMIM genes" are considered well studied, "cancer genes" have distinct PPI network features from other disease genes.

### Enrichment of protein domains and GO categories

We investigated the difference in the enrichment of various protein domains from the Pfam Database [20] and GO annotations [25] between cancer genes and non-cancer genes. Chi-square tests were conducted for each Pfam domain and GO term regarding the number of cancer genes assigned with the annotation compared to non-cancer genes. Significantly over-represented 'molecular function' terms in cancer genes include protein tyrosine kinase activity, DNA binding, and transcription regulator activity. 'biological process' terms significantly over-represented in cancer genes include negative regulation of cell cycle, response to DNA damage stimulus, and protein amino acid phosphorylation (Table 1). Most under-represented GO terms in cancer genes are ion transport and transporter activity. In agreement with GO term analysis, Pfam domain analysis shows that protein tyrosine kinase domain is most significantly enriched in cancer genes, followed by kinase domain, ets domain, paired box domain, DEAD box helicase and DNA mismatch repair domain (Table 2).

**Table 1:** Top 5 significantly over-represented GO terms of 'molecular function' and 'biological process' in cancer genes vs. non-cancer genes

**GO_ID**	**# cancer genes**	**# non-cancer genes**	**chi-square**	**p-value**	**Name**	**Name space**
45786	35	243	260.6	1.30E-58	negative regulation of cell cycle	biological_process

6974	34	244	259.4	2.30E-58	response to DNA damage stimulus	biological_process

50794	181	97	238.8	7.20E-54	regulation of cellular process	biological_process

9719	35	243	235.2	4.29E-53	response to endogenous stimulus	biological_process

6468	42	236	232.8	1.49E-52	protein amino acid phosphorylation	biological_process

4713	25	253	280.7	5.37E-63	protein-tyrosine kinase activity	molecular_function

3677	122	156	195.7	1.82E-44	DNA binding	molecular_function

4672	38	240	193.8	4.78E-44	protein kinase activity	molecular_function

30528	95	183	173.0	1.60E-39	Transcription regulator activity	molecular_function

16733	39	239	154.8	1.54E-35	phosphotransferase activity, alcohol group as acceptor	molecular_function

**Table 2:** Top 10 significantly over-represented Pfam domains in cancer genes vs. non-cancer genes

**ID**	**# cancer genes**	**# non-cancer genes**	**Chi-square**	**p-value**	**Name**	**Description**
PF07714	25	21	245.2	2.88E-55	Pkinase_Tyr	Protein tyrosine kinase

PF00069	31	54	188.2	8.03E-43	Pkinase	Protein kinase domain

PF00178	7	0	122.1	2.17E-28	Ets	Ets-domain

PF00292	4	0	61.5	4.36E-15	PAX	'Paired box' domain

PF00270	7	10	43.0	5.42E-11	DEAD	DEAD/DEAH box helicase

PF01119	3	0	41.6	1.11E-10	DNA_mis_repair	DNA mismatch repair protein, C-terminal domain

PF02518	5	4	41.5	1.18E-10	HATPase_c	Histidine kinase-, DNA gyrase B-, and HSP90-like ATPase

PF00017	9	20	39.6	3.08E-10	SH2	SH2 domain

PF00855	5	5	36.5	1.53E-09	PWWP	PWWP domain

PF00046	17	90	28.2	1.11E-07	Homeobox	Homeobox domain

**Figure 2:**
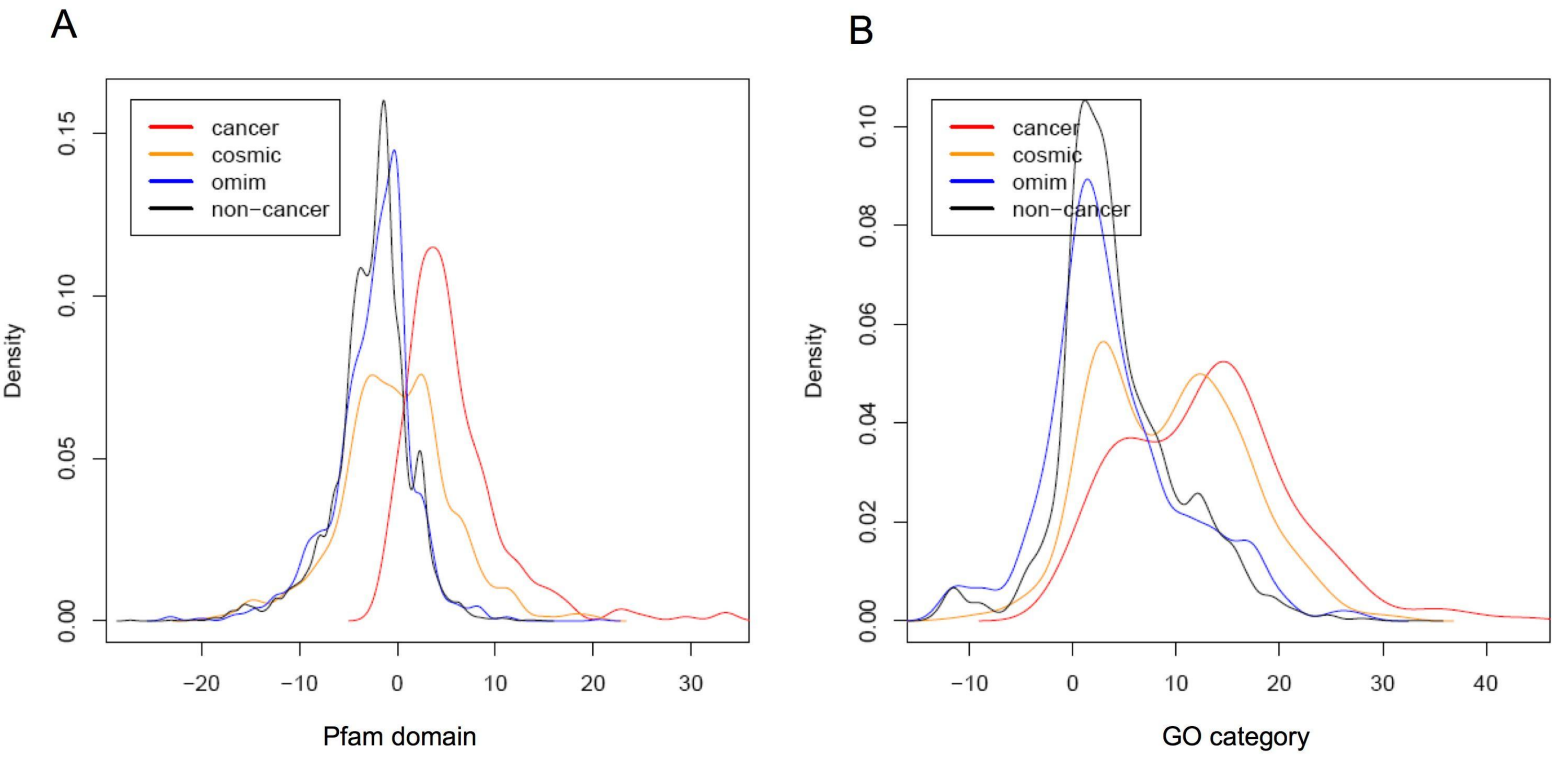
**Probability density distributions of log-odds scores of Pfam and GO enrichment in cancer genes**. A) Pfam B) GO.

We then compared the patterns of Pfam and GO annotations among cancer, COSMIC, OMIM and non-cancer gene groups. We calculated the log-odds ratio for each Pfam domain and GO slim term measuring the differential frequency of being assigned to cancer genes vs. non-cancer genes. GO slim was used because it gives a broad overview of GO ontology content with reduced details. A gene-based log-odds score was subsequently derived as the sum of log-odds ratios for each Pfam domain and GO term assigned to a given gene [14]. Overall, the probability density distributions of log-odds scores calculated using Pfam domain annotation is similar to that calculated using GO slim term annotation for each given gene set. The probability density distributions of log-odds scores are remarkably different between cancer genes and non-cancer genes for both GO and Pfam annotations (Figure 2). Notably, COSMIC genes show a bi-mode distribution where the first mode residing in similar position with the non-cancer genes while the second mode residing in similar position with the cancer genes. The bi-mode distribution suggests that a subset of COSMIC genes show similar domain composition and GO annotations as the cancer gene, while the rest show similarity to the non-cancer genes. Overall, COSMIC genes have larger log-odds score than non-cancer genes and OMIM genes, and OMIM genes are similar to non-cancer genes.

### Sequence features

We studied four sequence related features including protein length, gene length, the number of exons and the number of alternative transcripts (Table 3). Cancer genes have greater protein length than each of the other gene groups (*t *test p-value < 0.001). The median protein length for cancer genes is 591 aa, compared with the median protein length of 487 aa in COSMIC genes, 498 aa in OMIM disease genes and 433 aa in non-cancer genes. With respect to total gene length (including the length of introns and un-translated regions), the median gene length is 51 kb for cancer genes, 31 kb for COSMIC genes, 27.5 kb for OMIM genes and 21.7 kb for non-cancer genes; the median gene length of cancer genes are over two times longer than that of non-cancer genes (*t *test p-value < 0.01). OMIM genes are also longer than non-cancer genes (*t *test p-value < 0.01); it was previous shown that disease genes on average are longer than house-keeping genes and other genes in the genome [28]. Interestingly, genes involved in cancer, a complex disease, are on average longer than other disease genes that do not overlap with cancer or COSMIC genes. Assuming a constant mutation rate per nucleotide, longer genes are more susceptible to mutation that may lead to either disruption or activation of gene function.

**Table 3:** Summary statistics of sequence features.

**Features**	**cancer genes**	**non-cancer genes**	**COSMIC genes**	**OMIM genes**	**P-value**^**c**^
Protein sequence length (aa)	761 ± 25^a^ (591)^b^	557 ± 22 (433)	634 ± 26 (487)	713 ± 30 (498)	1e-11

Genomic sequence length (bp)	84163 ± 330 (51082)	54319 ± 317 (21676)	66471 ± 322 (30953)	63604 ± 330 (27515)	4.42e-11

Number of exons	10.24 ± 2.66	10.01 ± 2.98	10.49 ± 3.24	10.39 ± 2.97	0.096

Number of alternative splicing events	1.82 ± 1.23	1.36 ± 0.97	1.50 ± 0.99	1.54 ± 1.07	1e-11

^a ^mean and standard deviation

^b ^median

^c ^from ANOVA F-test

With regards to other sequence features we examined, the probability density distributions did not show clear separation between cancer and non-cancer genes regarding the average number of alternative transcripts (data not shown); no significant difference was observed in the number of exons among the four gene groups (ANOVA *F *test p-value 0.096).

### Evolutionary conservation

**Figure 3:**
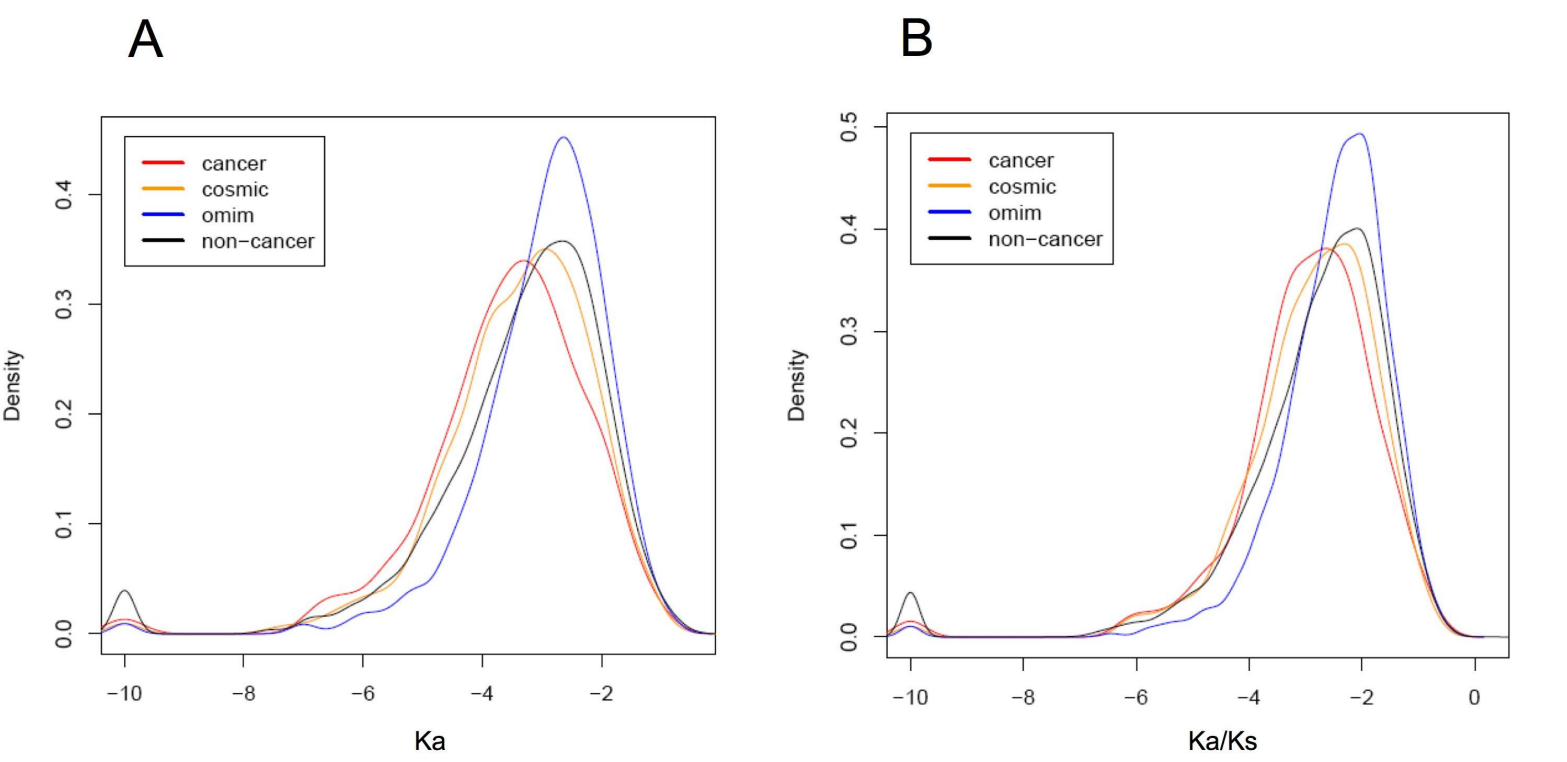
**Probability density distributions of Ka and Ka/Ks (log-transformed)**. A) Ka B) Ka/Ks.

The non-synonymous mutation rate (Ka) and the ratio of non-synonymous mutation rate (Ka) over synonymous rate (Ks) [29] between human-mouse and human-rat orthologs were compared among cancer, COSMIC, OMIM and non-cancer genes (Figure 3; see additional file 1: Supplementary Table S2). Ka measures how fast a given protein evolves, while the ratio between Ka and Ks is an important metric for selection pressure; smaller Ka/Ks value indicates that the gene is more conserved and may play an essential cellular function. Notably, cancer genes show significantly smaller Ka and Ka/Ks compared to non-cancer genes and OMIM genes (p-value < 0.001), indicating cancer genes evolve relatively slowly and are more conserved between human and rodent species. COSMIC genes have more similar Ka and Ka/Ks values to cancer genes rather than non-cancers.

### Construction of classifiers

As distinctive patterns were observed between cancer and non-cancer genes from the analyses of PPI networks, annotations of GO and Pfam, sequence and conservation features, we sought to design a classifier to combine the predictive power of each type of feature for identification of cancer genes. Specifically, we considered PPI network features including degree, clustering coefficient and the length of the shortest path to a cancer gene, sequence features including gene and protein lengths, and conservation features including Ka and Ka/Ks ratio. For GO and Pfam features, we selected 79 GO terms and 61 Pfam domains significantly differentially represented in caner genes compared to non-cancer genes by chi-square test (p < 0.01). For a given gene, presence and absence of assignment of each GO term or Pfam domain was encoded as '1' or '0' respectively.

**Figure 4:**
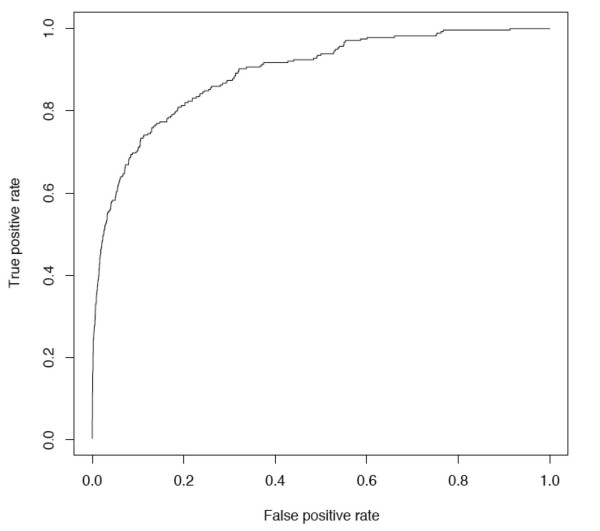
**ROC curve of SVM classifier**.

Several machine-learning algorithms were investigated in building a classifier to predict novel cancer genes, including naïve Bayes, logistic regression and support vector machines (SVM), all of which have been widely used for pattern classification and regression problems. Naïve Bayes is a simple probabilistic classifier based on Bayes rules and has a strong assumption that the features are independent to each other. Logistic regression is a model used for prediction of the probability of occurrence of an event by fitting data to a logistic curve. SVMs map input examples to a higher dimensional feature space using a kernel function, and identify a separating hyperplane that maximizes the margin or distance from the hyperplane to the nearest positive and negative examples [30]. Classifiers based on SVM, naïve Bayes and logistic regression methods were trained with the "cancer gene" group as positive examples and the "non-cancer gene" group as negative examples. We built the classifier using different types of features both individually and in combination and evaluated the predictive power of each classifier by 10-fold cross validation experiments (Table 4). The performance is measured by Receiver Operating Characteristic (ROC) curves [31], which plot the true positive rates against false positive rate at various thresholds (Figure 4). The area under ROC curve (AUC) provides the metric of overall performance of the classifier. Among SVMs using different types of features individually, GO annotation gives the best performance with AUC of 0.830, followed by PPI (AUC 0.767) and Pfam (AUC 0.706) features. Combining PPI features with GO and Pfam features gives an AUC of 0.886 and increases the performance by 6.7% - 25.5% compared to their individual performances. In contrast, sequence and conservation features have relatively weak predictive power (AUC less than 0.6). Different classification methods have similar performance when the number of features is small, i.e. when using each type of features alone. The classifier of SVMs combining PPI, GO, Pfam, sequence and conservation features gives the best performance (AUC 0.896) of all method-feature selections being evaluated. Adding PPI features in building the SVM classifier resulted 4.2% increase in AUC compared to the SVM using all the other features. The ROC curve shows that the specificity (1 - false positive rate) is 80% when the sensitivity (true positive rate) is 82% (Figure 4).

**Table 4:** Area under ROC (AUC) for feature selections and classifiers

**Feature selection**	**SVM**	**Naïve Bayes**	**Logistic regression**
PPI	0.767	0.758	0.773

GO	0.830	0.824	0.806

Pfam	0.706	0.697	0.703

Sequence (gene + protein length)	0.592	0.619	0.618

Conservation (Ka + Ka/Ks)	0.580	0.571	0.591

GO + Pfam	0.848	0.826	0.826

GO + Pfam + Sequence	0.858	0.829	0.831

GO + Pfam + Conservation	0.850	0.826	0.828

GO + Pfam + Sequence + Conservation	0.860	0.829	0.837

PPI + GO + Pfam	0.884	0.843	0.858

PPI + GO + Pfam + Sequence	0.892	0.846	0.859

PPI + GO + Pfam + Conservation	0.886	0.843	0.859

PPI + GO + Pfam + Sequence + Conservation	0.896	0.846	0.861

### Application of the SVM classifier

**Figure 5:**
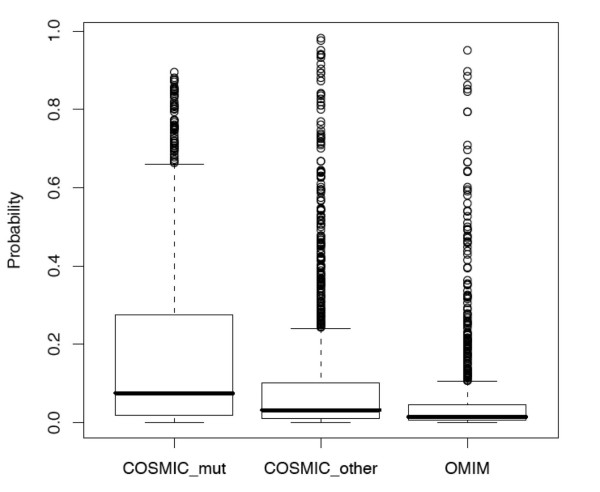
**Probability scores predicted by SVM classifier for COSMIC and OMIM genes**.

We applied the SVM classifier that was trained using cancer and non-cancer gene groups to COSMIC and OMIM gene groups, which were held out from the training set, to estimate their probabilities of being involved in cancer (Figure 5). Among the COSMIC gene set, somatic mutations were found from at least one sample in 805 genes (noted as "COSMIC_mut") whereas no mutations were found in 1386 genes (noted as "COSMIC_other") based on the COSMIC database. Overall COSMIC_mut genes have higher probability scores than COSMIC_other genes (*t *test p-value 4.3e-18) and OMIM genes (*t *test p-value 2e-41); COSMIC_other genes have higher probability scores than OMIM genes (*t *test p-value 6.5e-13). Specifically, 102 out of 805 COSMIC_mut genes, 72 out of 1386 COSMIC_other genes, and 25 out of 1088 OMIM genes have a probability score no less than 0.5 (see additional file 1: Supplementary Table S3).

### siRNA experiments

RNAi-based phenotypic screening has demonstrated its utility in identifying cancer genes and putative drug targets [32-34]. As genes that are essential for cancer cell proliferation and survival represent attractive drug target candidates, we examined a subset of predicted cancer genes using small interference RNA (siRNA) knockdown and cell viability assays. Although our predictions do not distinguish between oncogenes and tumor suppressor genes, we are interested in identifying novel oncogenes in this experiment for potential new therapeutics. As COSMIC, OMIM and non-cancer gene sets may contain novel oncogenes that have not been characterized as cancer genes in the Cancer Gene Census, we focused on COSMIC, OMIM and non-cancer genes and examined whether their siRNA knockdown would lead to decreased viability of the cell. The phenotype of decreased viability when a gene is knocked down indicates that the gene is essential for cancer cell proliferation and may potentially become a novel drug target. A total of 332 from these three gene sets overlap with the duplex siRNA library for druggable genes (Dharmacon Inc.) and were included in a large siRNA screen conducted at our institution for druggable genes that affect the viability of human colon cancer cell line DLD-1 (unpublished). Among these, 16 genes are likely to be cancer genes having probability scores greater than 0.5 from the classifier (noted as predicted cancer genes) and the rest 316 genes are less likely to be involved in cancer (noted as predicted non-cancer genes). A viability score is defined as the ratio of cell viability after the transfection of the testing siRNA over the negative control siRNAs (non-targeting siRNAs). A viability score significantly less than 1 indicates that siRNA knockdown of the target gene significantly decreased cell viability. We conducted one-sample *t *tests of the viability scores from two replicated experiments for each of the four different siRNA oligos targeting the same gene. To identify genes whose siRNA knockdown leads to decreased viability in a cell line, we require that at least two of the four siRNAs targeting this gene produced significantly reduced viability (p < 0.05) and the decrease in cell viability is at least 15%. As a result, 6 out of the 16 (37.5%) predicted cancer genes vs. 40 out of the 316 (12.7%) predicted non-cancer genes were selected. Fisher's exact test showed a significant enrichment of genes essential for cell viability in predicted cancer genes vs. non-cancer genes (odds ratio 4.11 and p-value 0.014).

**Figure 6:**
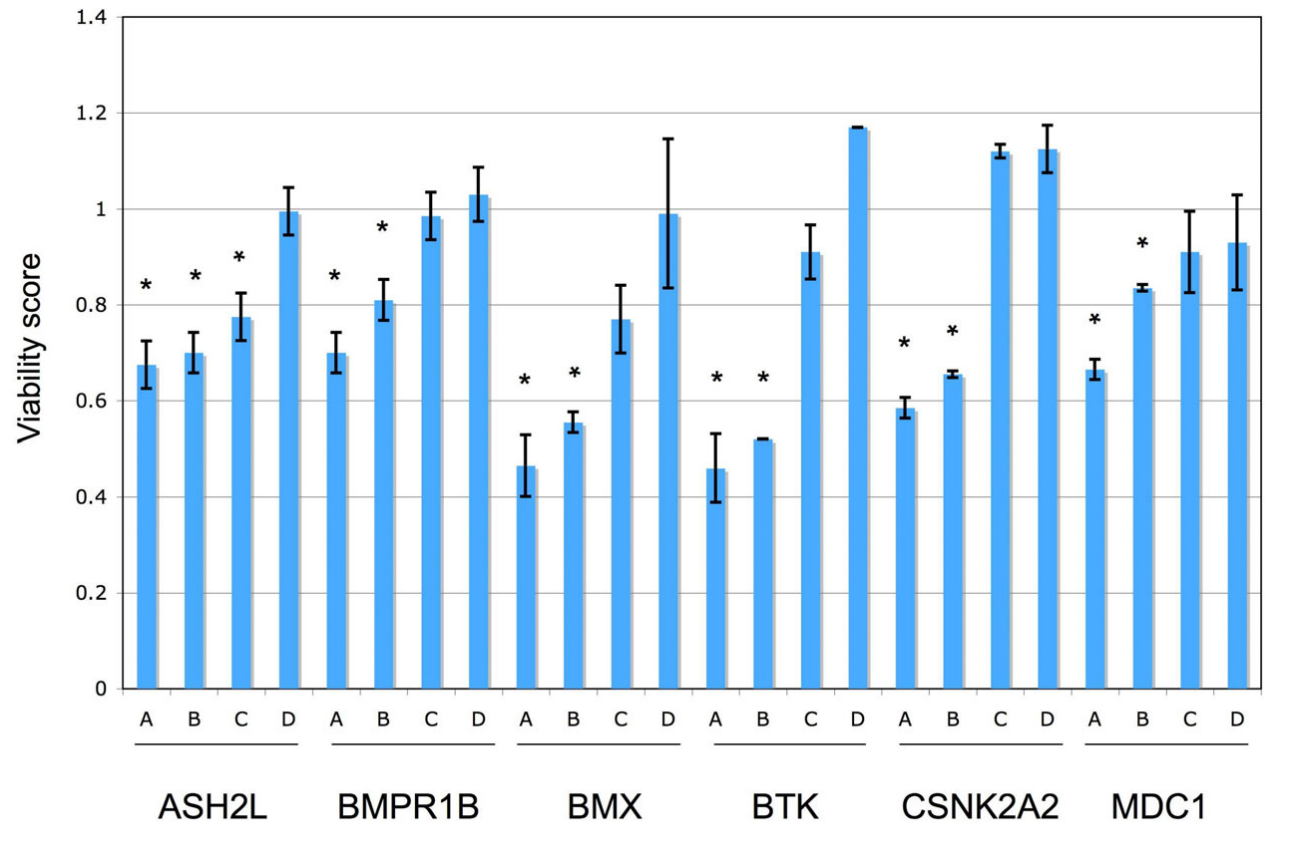
**Cell viability scores of candidate cancer genes in DLD-1**. Four siRNA oligos targeting the same gene are labeled as 'A', 'B', 'C' and 'D' for each gene. Each bar shows the mean and standard deviation of the viability scores of two replicated experiments for each siRNA. siRNAs showing significantly decreased viability (p < 0.05) are labeled with '*'.

Figure 6 shows the viability scores of the six candidate cancer genes whose siRNA knockdown resulted in decreased cell viability in DLD-1, including ASH2L, BMX, BMPR1B, BTK, CSNK2A2 and MDC1. Among the 24 siRNAs from these six genes, 13 caused significant reduction of cell viability in DLD-1 (p < 0.05, noted by '*' in Figure 6). BMX, BMPR1B, BTK, and MDC1 belong to the COSMIC_mut gene set, and CSNK2A2 belongs to the COSMIC_other gene set, while ASH2L belongs to the non-cancer gene set. BMX, a non-receptor tyrosine kinase, is involved in cell adhesion, migration, and survival in tumor necrosis factor (TNF)-induced angiogenesis [35]. It was shown to interact with Pim-1 kinase and be required for ligand-independent activation of androgen receptor in prostate cancer [36]. BTK, Bruton tyrosine kinase, is associated with agammaglobulinemia, an X-linked immunodeficiency that involves a failure in normal development of B lymphocytes, via missense mutations [37]. MDC1, mediator of DNA damage checkpoint 1, mediates transduction of the DNA damage signal and controls damage-induced cell-cycle arrest checkpoints [38]. CSNK2A2, alpha subunit of casein kinase 2 (CK2), is a ubiquitous and pleiotropic Ser/Thr protein kinase involved in cell growth and transformation. Deregulated expression of this kinase was observed in tumors of the prostate, kidney, colon and squamous cell carcinoma of the head and neck. Recent evidence points to CK2 as an important regulator of apoptosis pathways, suggesting that CK2 might be an important target for cancer therapy [39]. ASH2L (absent, small, or homeotic discs 2-like) is the human homolog of a Drosophila gene that was found to be involved in the segmentation of the embryo [40]. Interestingly, human ASH2L was found to interact with oncoprotein MLL and MYC [41,42]. Although ASH2L was originally included in the "non-cancer" gene set, a positive prediction from our cancer gene classifier and observed phenotypes from siRNA experiments indicate that ASH2L might be a novel cancer gene. In fact, a very recent study showed that ASH2L has transforming activity in rat embryo fibroblast and cooperates with activated Ha-RAS, suggesting ASH2L is a novel oncogene [43]. This analysis suggests that our classifier can potentially prioritize "hits" generated from large-scale functional genomic assays and propose novel candidates for further investigations. We anticipate application of our cancer gene predictions to future whole genome RNAi screens in a larger collection of cancer cell lines can potentially reveal novel therapeutic targets.

## Discussion

Our study represents a first attempt to examine the predictive power of PPI network properties, in combination with an extensive set of structural and functional features, for identification of cancer genes. Compared to OMIM disease genes and non-cancer genes, cancer genes have more interaction partners, higher network density in their neighborhood, and are more closely related to other cancer genes in the PPI network. These observations agree with the notions that cancer genes play a central role in the cellular network and exert functions in an inter-dependant modular fashion. One common concern regarding analysis of PPI network is that the observed higher connectivity of certain group of genes could be a result of a bias in the PPI network, as it could be argued that these genes received more detailed investigations by the research community. To address this concern, it was previously argued that higher number of known interaction partners for cancer genes is likely to be a consequence of higher frequency of promiscuous domains (which interact with a variety of different domains) in caner genes rather than obvious bias in the PPI network [8]. Based on a probability density function from the Pfam domain population [8], many of the top Pfam domains enriched in cancer genes vs. non-cancers in our study showed significantly higher-than-expected interaction promiscuity in term of the number of different domains they interact with, such as protein kinase domain, Ets domain and Homeobox domain (Table 2). In addition, there is significant difference in connectivity and clustering coefficient between cancer and OMIM genes (Figure 1; see additional file 1: Supplementary Table S1) even though cancer genes and OMIM genes both represent heavily studied gene sets. ~90% of both cancer genes from Cancer Gene Census and disease genes from OMIM database were included in the PPI network. Furthermore, the analyses were conducted using the subset of well-annotated genes from human genome that were assigned with GO terms and Pfam domains. As a result, the less well-studied genes were filtered out from the non-cancer gene group.

Our study showed that cancer genes have distinctive functional, sequence and evolutionary characteristics from COSMIC, OMIM and non-cancer genes. COSMIC genes and OMIM genes in turn have distinctive features between each other and from non-cancer genes. It should be noted that the OMIM gene set in our study is specific to the context of comparison with cancer genes as we excluded from the OMIM gene set those common between the OMIM database and Cancer Census Genes or COSMIC database. COSMIC genes showed relatively more similarities with cancer genes in many properties, and in fact many COSMIC genes were found to be involved in cancer although they are not included in the Cancer Gene Census database [15]. Therefore, it is beneficial to separate COSMIC and OMIM gene groups from non-cancer genes in training a classifier to predict cancer genes.

SVM classifiers on average perform slightly better than Naïve Bayes and logistic regression. Naïve Bayes performs the worst in our study probably due to the fact that our feature vectors are not orthogonal to each other, which violated the basic assumption of Naïve Bayes models. The theoretical advantage of SVMs is that they simultaneously minimize the empirical classification error and maximize the geometric margin; the idea of maximizing the margin mitigates the problem of over-fitting the training data, which is of particular importance when dealing with large number of features.

PPI topological features alone have relatively strong predictive power for identification of cancer genes. Similar to PPI features, GO and Pfam annotations are strong predictors compared to sequence and conservation features. Combining all these features maximize the predictive power (Table 4). With the accumulation of more and more protein-protein interaction datasets, our approach of integrating PPI topological features will potentially become more powerful in the future.

The SVM classifier provides a probability score to prioritize candidate cancer genes, which can be followed up by experimental studies, such as siRNA knock down and cell viability assays. Preliminary siRNA studies on predicted cancer genes showed promising leads for further investigations. Interestingly, COSMIC genes with somatic mutations in cancer samples have higher scores than other genes in the COSMIC database (Figure 5). As COSMIC genes were held out from the training set and no mutation information was included in the training features, this observation indicates our approach aligns with the large-scale systematic re-sequencing efforts and can serve as a useful complementary approach for identifying cancer genes.

## Conclusion

Topological features of PPI networks, protein domain compositions and GO annotations are good predictors of cancer genes. The SVM classifier integrates multiple features and as such is useful for prioritizing candidate cancer genes for experimental validations. Preliminary siRNA studies on predicted cancer genes showed promising leads for further investigations. The integrative approach using PPI networks is a useful complement to large-scale systematic re-sequencing and other genomic discovery projects for identifying novel cancer genes.

## Abbreviations

PPI: protein-protein interaction; GO: Gene Ontology; SVM: support vector machine; COSMIC: Catalogue Of Somatic Mutations In Cancer; OMIM: Online Mendelian Inheritance in Man; BIND: Biomolecular Interaction Network Database; HPRD: Human Protein Reference Database; KEGG: Kyoto Encyclopedia of Genes and Genomes; ROC: Receiver Operating Characteristic; RBF: radial basis function.

## Competing interests

The authors declare that they have no competing interests.

## Authors' contributions

ZT and LL designed the study. LL and KZ collected the data, performed the data analysis and drafted the manuscript. JL, SC and DD performed the siRNA experiments and prepared the corresponding Method sections. All authors read and approved the final manuscript.

## Pre-publication history

The pre-publication history for this paper can be accessed here:



## Supplementary Material

Additional file 1 **Supplementary Table S1-S3**. Supplementary Table S1 Comparisons of network topological features among gene groups. Supplementary Table S2 Comparisons of Ka and Ka/Ks among gene groups. Supplementary Table S3 COSMIC and OMIM genes having probability greater than 0.5 from the SVM classifier. Click here for file
